# Production of mycobacterial cell wall glycopeptidolipids requires a member of the MbtH-like protein family

**DOI:** 10.1186/1471-2180-12-118

**Published:** 2012-06-22

**Authors:** Elizabeth Tatham, Sivagami sundaram Chavadi, Poornima Mohandas, Uthamaphani R Edupuganti, Shiva K Angala, Delphi Chatterjee, Luis E N Quadri

**Affiliations:** 1 Department of Biology, Brooklyn College, City University of New York, 2900 Bedford Avenue, Brooklyn, NY 11210, USA; 2 Department of Microbiology, Immunology and Pathology, College of Veterinary Medicine and Biomedical Sciences, Colorado State University, 1682 Campus Delivery, Fort Collins, CO 80523, USA

## Abstract

**Background:**

Glycopeptidolipids (GPLs) are among the major free glycolipid components of the outer membrane of several saprophytic and clinically-relevant *Mycobacterium* species. The architecture of GPLs is based on a constant tripeptide-amino alcohol core of nonribosomal peptide synthetase origin that is *N*-acylated with a 3-hydroxy/methoxy acyl chain synthesized by a polyketide synthase and further decorated with variable glycosylation patterns built from methylated and acetylated sugars. GPLs have been implicated in many aspects of mycobacterial biology, thus highlighting the significance of gaining an understanding of their biosynthesis. Our bioinformatics analysis revealed that every GPL biosynthetic gene cluster known to date contains a gene (referred herein to as *gplH*) encoding a member of the MbtH-like protein family. Herein, we sought to conclusively establish whether *gplH* was required for GPL production.

**Results:**

Deletion of *gplH*, a gene clustered with nonribosomal peptide synthetase-encoding genes in the GPL biosynthetic gene cluster of *Mycobacterium smegmatis*, produced a GPL deficient mutant. Transformation of this mutant with a plasmid expressing *gplH* restored GPL production. Complementation was also achieved by plasmid-based constitutive expression of *mbtH*, a paralog of *gplH* found in the biosynthetic gene cluster for production of the siderophore mycobactin of *M. smegmatis*. Further characterization of the *gplH* mutant indicated that it also displayed atypical colony morphology, lack of sliding motility, altered capacity for biofilm formation, and increased drug susceptibility.

**Conclusions:**

Herein, we provide evidence formally establishing that *gplH* is essential for GPL production in *M. smegmatis*. Inactivation of *gplH* also leads to a pleiotropic phenotype likely to arise from alterations in the cell envelope due to the lack of GPLs. While genes encoding MbtH-like proteins have been shown to be needed for production of siderophores and antibiotics, our study presents the first case of one such gene proven to be required for production of a cell wall component. Furthermore, our results provide the first example of a *mbtH*-like gene with confirmed functional role in a member of the *Mycobacterium* genus. Altogether, our findings demonstrate a critical role of *gplH* in mycobacterial biology and advance our understanding of the genetic requirements for the biosynthesis of an important group of constituents of the mycobacterial outer membrane.

## Background

The cell envelope of members of the *Mycobacterium* genus contains a unique array of structurally-complex free lipids thought to be non-covalently bound to the mycolic acid layer of the cell wall [1-3]. These free lipids are believed to form a membrane outer leaflet that partners with a mycolic acid-based membrane inner leaflet to form an asymmetric lipid bilayer-like structure. This lipid bilayer constitutes the distinctive outer membrane of the mycobacterial cell envelope. The documented role of some of these free lipids as mycobacterial virulence effectors highlights the enzymes involved in their production as potential target candidates for exploring the development of novel drugs that could assist conventional antimicrobial therapy in the control of mycobacterial infections. Notably, the first inhibitor of the biosynthesis of a group of these free lipids (*i.e*., phenolic glycolipids [3]) has been recently reported [4]. The inhibitor works in a manner analogous to that of the first reported inhibitor of siderophore (iron chelator) biosynthesis [5,6], and it blocks the production of phenolic glycolipids in *Mycobacterium tuberculosis* and other mycobacterial pathogens [4].

**Figure 1:**
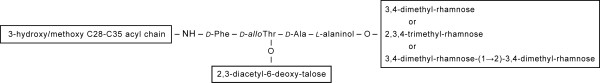
**Representative structures of glycopeptidolipids.** The depicted GPLs correspond to those found in *Mycobacterium smegmatis*.

Glycopeptidolipids (GPLs) are among the major free glycolipid components of the outer membrane of several *Mycobacterium* species [7,8] (Figure 1). The GPL-producing species include saprophytic mycobacteria, such as *Mycobacterium smegmatis* (*Ms*), and many clinically-relevant nontuberculous mycobacteria. The members of the *Mycobacterium avium-Mycobacterium intracellulare* complex (MAC) are among the GPL producers of clinical significance. MAC infections cause pulmonary and extrapulmonary diseases in both immunocompromised and immunocompetent individuals [9,10]. Importantly, GPLs have been implicated in many aspects of mycobacterial biology, including host-pathogen interaction [11-17], sliding motility [18,19], and biofilm formation [18,20]. An altered expression profile of GPLs has been observed in drug-resistant clinical isolates of MAC [21], a finding that raises the possibility that GPL production might have an impact on drug susceptibility as well. Thus, elucidation of the GPL biosynthetic pathway is important not only because it will expand our understanding of cell wall biosynthesis in mycobacteria, but it may also illuminate potential routes to alternative therapeutic strategies against infections by MAC and other opportunistic mycobacterial human pathogens.

Structures of GPLs from several mycobacteria have been characterized (reviewed in references [7,8]). In brief, GPL molecules are composed of an *N*-acylated lipopeptide core decorated by a variable pattern of glycosylation that is built from *O*-methylated and *O*-acetylated sugar units. The peptide moiety is the tripeptide-amino alcohol D-phenylalanine-D-*allo*threonine-D-alanine-L-alaninol (D-Phe-D-*allo*Thr-D-Ala-L-alaninol). This tripeptide-amino alcohol is assembled by nonribosomal peptide synthetases (NRPSs) designated Mps1 and Mps2 in *Ms*[22-25], whereas biosynthesis of the lipid substituent (3-hydroxy/methoxy C28-C35 acyl chain) is believed to require a dedicated polyketide synthase (PKS) [24]. NRPSs and PKSs are two large families of enzymes that are best known for their involvement in the synthesis of natural products with pharmacological activities of clinical significance [26,27] and microbial siderophores [28,29]. *N*-acylation of the tripeptide-amino alcohol of *Ms* GPLs has been proposed to require the protein PapA3 [24], a member of the polyketide-associated protein (Pap) family of acyltransferases [30,31]. Lastly, various glycosyltransferases, methyltransferases and acetyltransferases have been implicated or are suspected to be involved in the building of the glycosyl portion of GPLs [7,8,24,32].

Despite the increasingly recognized widespread presence of GPLs in mycobacteria and the relevance of these compounds in MAC and other mycobacteria of clinical significance, the GPL biosynthetic pathway remains incompletely understood. The individual involvement of several genes suspected to be required for GPL production remains to be experimentally probed. In particular, the involvement of a gene encoding a member of the MbtH-like protein family (NCBI CDD pfam 03621) [33,34] and clustered with the NRPS-encoding genes required for D-Phe-D-*allo*Thr-D-Ala-L-alaninol assembly in GPL production has been hypothesized [23-25,35], but not conclusively demonstrated. MbtH-like proteins form a family of small proteins (60–80 amino acids) linked to secondary metabolite production pathways involving NRPSs [34]. The founding member of this protein family is MbtH, a protein encoded in the mycobactin siderophore biosynthetic gene cluster of *M. tuberculosis*[33].

Recent seminal biochemical studies have established that MbtH-like proteins activate amino acid adenylation domains of NRPSs [36-40]. Genes encoding MbtH-like proteins have been shown to be required for production of siderophores or antibiotics by mutational analysis [41-44]. Interestingly, however, we have recently shown by mutational analysis that the *mbtH* orthologue in the mycobactin biosynthetic gene cluster of *Ms* (MSMEG_4508) is not essential for mycobactin production [35]. Similarly, the *mbtH*-like gene in the biosynthetic gene cluster of the balhimycin glycopeptide antibiotic has been shown not to be required for antibiotic production [45]. These findings illustrate the well-recognized limitations of bioinformatics-based genetic predictions in complex biosynthetic pathways and highlight the need for probing gene involvement by unconfounded mutational analysis. In this study, we provide evidence unequivocally establishing that the conserved *mbtH*-like gene (herein referred to as *gplH*) located in the GPL biosynthetic gene locus of *Ms* is essential for GPL production. This finding presents the first case of a *mbtH*-like gene required for biosynthesis of a cell wall component and provides the first example of a *mbtH*-like gene with confirmed functional role in a member of the *Mycobacterium* genus. Moreover, we show that loss of *gplH* leads to a mutant with atypical colony morphology, lack of sliding motility, reduced biofilm formation capacity, and increased antimicrobial drug susceptibility. Altogether, this study demonstrates a critical role for *gplH* in mycobacterial biology and advances our understanding of the genetic requirements for the biosynthesis of an important group of constituents of the unique mycobacterial outer membrane.

## Results and discussion

### Conservation of a MbtH homologue in the GPL biosynthetic pathway

**Figure 2:**
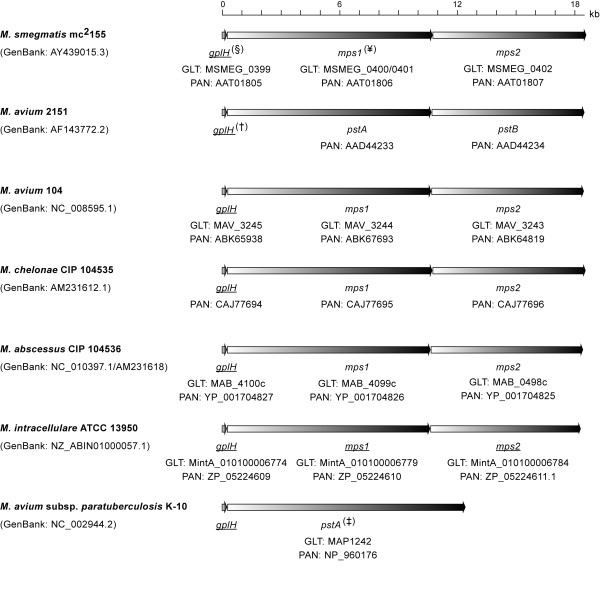
**Orthologous *mbtH*-like gene (*gplH*)-nonribosomal peptide synthetase gene loci involved in GPL production.**^§^ Nucleotide sequence accession numbers (GenBank) of analyzed sequences, protein accession numbers (PAN), available genomic locus tags (GLT), and gene names are shown. Underlined gene names are proposed herein. ^¥^*M. smegmatis* sequence submission AY439015.3 shows a single gene (*mps1*) where the annotated complete genome (GenBank: CP000480.1) shows two contiguous genes (MSMEG_0400 and MSMEG_0401). Our sequence comparison revealed that CP000480.1 has an insertion of a “C” and a deletion of an “A” relative to AY439015.3. The events (112-bp apart) create a transient frameshift that splits *mps1* into MSMEG_0400 and MSMEG_0401. We resequenced the region containing the discrepancies and found that our sequence matched that of AY439015.3. Based on this and the conservation of *mps1* across species, we conclude that the correct gene organization is as shown herein. ^†^The open reading frame corresponding to this gene has not been previously annotated. ^‡^Our sequence analysis (not shown) indicates that the *pstA* appears to have originated from an *mps1* and *mps2* deletion-fusion rearrangement relative to the canonical *mps1* and *mps2* seen in *M. avium* strains 104 and 2151 and other GPL-producing species. This rearrangement leads to a gene encoding a 4,027-amino acid protein that appears to have segments derived from both Mps1 and Mps2. This protein would not be competent for D-Phe-D-*allo*Thr-D-Ala-L-alaninol synthesis, a defect that alone would explain the known GPL-deficiency of *M. avium* subsp. *paratuberculosis* K-10.

**Figure 3:**
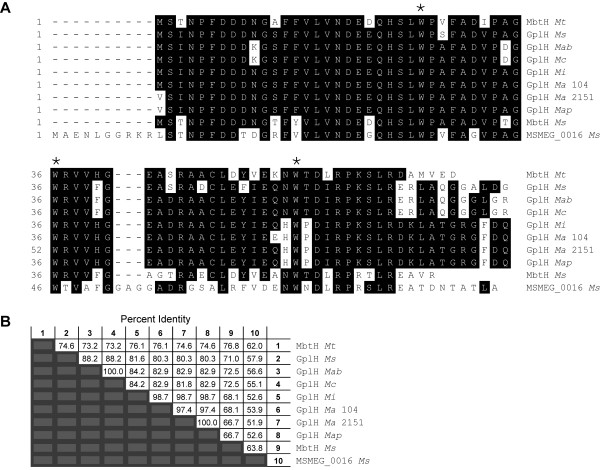
**Sequence relatedness of GplH orthologues and related homologues.** (**A**) Protein alignment and (**B**) table of percentage of amino acid identity. Conserved amino acids that match consensus are highlighted in white font over black background. The three conserved tryptophan residues that are the hallmark of the MbtH-like protein family are marked (*). The protein alignment and identity determination were performed with ClustalW (Lasergene software, DNASTAR, Inc). *Mab, M. abscessus; Ma, M. avium; Map, M. avium* subsp. *paratuberculosis; Mc, M. chelonae; Mi, M. intracellulare; Ms, M. smegmatis; Mt, M. tuberculosis*.

MbtH is a protein encoded in the mycobactin siderophore biosynthetic gene cluster of *M. tuberculosis* and the founding member of the MbtH-like protein family (NCBI CDD pfam 03621) [33]. Our analysis of available genome sequences of GPL producers revealed that every GPL biosynthetic gene cluster known to date contains a *mbtH*-like gene located upstream of NRPS-encoding genes required for D-Phe-D-*allo*Thr-D-Ala-L-alaninol assembly (Figure 2). The MbtH-like protein orthologues encoded by these *mbtH*-like genes are comprised of 69–93 amino acids and have remarkable sequence identity (80-100%) (Figure 3). This sequence identity extends to the three fully conserved tryptophan residues that are a hallmark of the protein family (NCBI CDD pfam 03621) [33] (Figure 3A). The open reading frame corresponding to the *mbtH*-like gene of *M. avium* 2151 (Figure 2) has not been previously annotated; however, our genome sequence analysis revealed its presence. The MbtH-like protein encoded by this gene is shown in the protein alignment (Figure 3A). The orthologous *mbtH*-like genes or MbtH-like proteins in the other species shown in Figure 2 have been annotated each as *mbtH* or MbtH, respectively [24,46], presumably due to their sequence relatedness with *M. tuberculosis* MbtH. This name assignment is misleading as these genes are not orthologues of *mbtH*, the gene of the mycobactin biosynthetic pathway present in many mycobacteria, including *M. smegmatis, M. abscessus*, and *M. avium*[33,35]. This name assignment leads to gene nomenclature confusion by resulting in more than one gene named *mbtH* in the same species. We proposed herein to name all the orthologous *mbtH*-like genes associated with GPL production as *gplH*, a name derived from *g*lyco*p*eptido*l*ipid and *mbtH* and not previously assigned to any mycobacterial gene.

### Deletion of *gplH* in *M. smegmatis*

**Figure 4:**
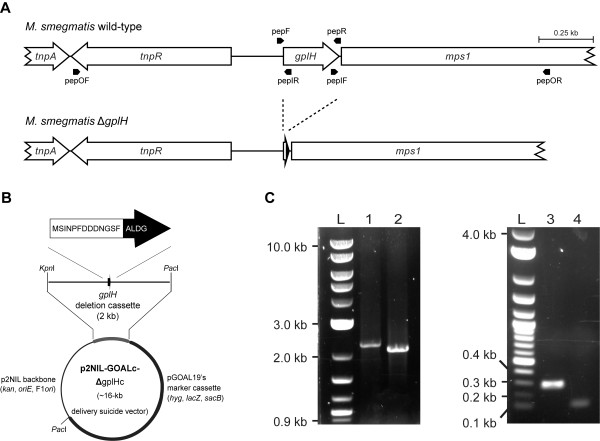
**Construction of *M. smegmatis *Δ*gplH.*** (**A**) Scheme illustrating the deletion in the chromosome of *Ms* Δ*gplH*. The position of each primer used in this study is shown. (**B**) Scheme of the *gplH* deletion cassette-delivery suicide vector used for construction of *Ms* Δ*gplH*. The *gplH* deletion leaves behind a gene remnant coding for only the first 13 (black print) and last 4 (white print) amino acids of GplH. This gene remnant in ΔgplHc is flanked by ~1 kb of downstream and upstream WT sequence for homologous recombination with the chromosome. (**C**) Agarose gel electrophoresis showing PCR-based confirmation of the *gplH* deletion in *Ms* Δ*gplH*. Lanes: 1, *Ms* WT (2,239-bp amplicon expected with primers pepOF and pepOR); 2, *Ms* Δ*gplH* (2,068-bp amplicon expected with primers pepOF and pepOR); 3, *Ms* WT (278-bp amplicon expected with primers pepF and pepR); 4, *Ms* Δ*gplH* (101-bp amplicon expected with primers pepF and pepR); L, DNA ladder marker.

Our bioinformatics analysis revealed that every GPL biosynthetic gene cluster known to date contains a *mbtH*-like gene, *gplH*. The involvement of this conserved gene in GPL production remains unproven. Herein, we sought to conclusively establish whether *gplH* was required for GPL production. To this end, we engineered *Ms* Δ*gplH*, a mutant with an unmarked, in-frame deletion of *gplH* (Figure 4A), the *Ms* gene upstream of the NRPS-encoding gene *mps1* (Figure 2), and assessed the ability of this mutant to produce GPLs as described below. *Ms* was selected as a representative prototype of GPL producers for the studies presented herein due to its superior experimental tractability compared with other GPL producers (*e.g*., MAC members).

The *gplH* deletion was engineered using the *gplH* deletion cassette-delivery suicide vector p2NIL-GOALc-ΔgplH c(~16 kb, Figure 4B) in a homologous recombination- and counter selection-based approach that replaced *gplH* by a 17-codon gene remnant cloned into the vector. The deletion in *Ms* Δ*gplH* encompassed 59 central amino acids of the predicted MbtH-like protein encoded by *gplH* (GplH, MSMEG_0399; 76 amino acids, Figure 3A). The deletion was verified by PCR using primer pairs that produced amplicons of different sizes depending on whether the genomic DNA used as PCR template was wild type (WT) or carried the gene deletion (Figure 4C). The successful engineering of *Ms* Δ*gplH* set the stage for probing the involvement of *gplH* in GPL production.

### The gene *gplH* is essential for GPL production

**Figure 5:**
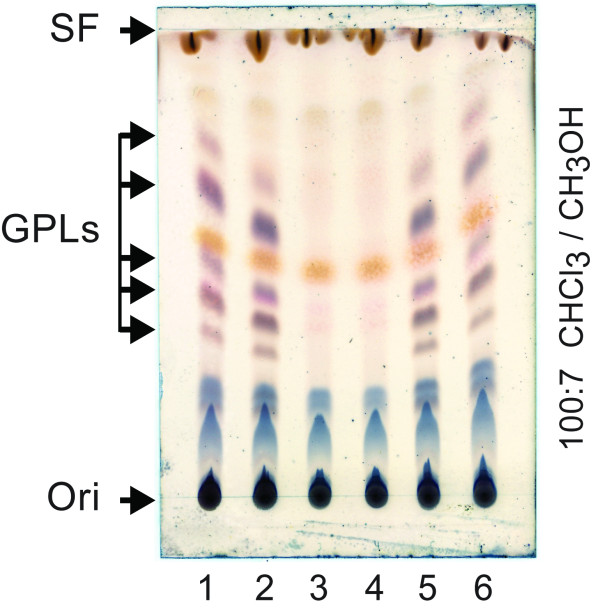
**Deletion of *gplH *leads to GPL deficiency.** Representative TLC analysis of lipid samples from: *Ms* WT; 2, *Ms* WT + pCP0; 3, *Ms* Δ*gplH*; 4, *Ms* Δ*gplH* + pCP0; 5, *M*s Δ*gplH* + pCP0-*gplH*; and 6, *Ms* Δ*gplH* + pCP0-*mbtH*Ms. The TLC solvent system is indicated. Ori, origin; SF, solvent front.

We investigated the effect of the *gplH* deletion in *Ms* Δ*gplH* on GPL production using TLC and MS analyses. In addition, a control strain for genetic complementation analysis was constructed (*Ms* Δ*gplH* + pCP0-*gplH*), and the ability of this strain and that of *Ms* WT controls to produce GPLs was investigated. Representative results from the TLC analysis are shown in Figure 5. The analysis of lipid extracts from the parental *Ms* WT strain, or *Ms* WT bearing the empty pCP0 vector, revealed the expected production of GPLs in these WT controls. Conversely, analysis of lipid extracts from *Ms* Δ*gplH* did not reveal detectable amounts of GPLs. Transformation of *Ms* Δ*gplH* with pCP0-*gplH* (a pCP0-based plasmid expressing *gplH*) rendered the strain *Ms* Δ*gplH* + pCP0-*gplH*, for which TLC analysis demonstrated that production of GPLs was restored to levels comparable to those seen in the WT controls. In contrast, *Ms* Δ*gplH* retained its GPL deficient phenotype after transformation with empty pCP0 vector (strain *Ms* Δ*gplH* + pCP0). The results of our complementation analysis rule out the possibility that the GPL deficient phenotype observed in *Ms* Δ*gplH* is due to a polar effect of the *gplH* deletion on downstream genes required for GPL production (*i.e., mps1* and *mps2*; Figure 2).

**Figure 6:**
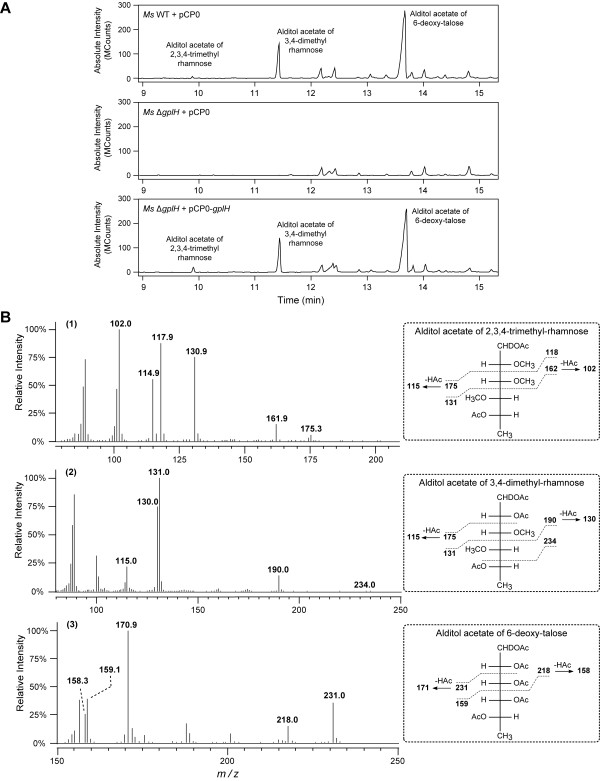
**GC-MS analysis of alditol acetate derivatives of the glycosyl residues of GPLs.** (**A**) Total ion count chromatographs displaying the presence or absence of alditol acetates in extracted lipid samples from the strains indicated. (**B**) Mass spectra showing fragmentation pattern fingerprints demonstrating alditol acetate identity in peaks labeled 2,3,4-trimethyl- rhamnose (1), 3,4-dimethyl-rhamnose (2), and 6-deoxy-talose (3) from *Ms* WT + pCP0. Equivalent spectra were observed for the samples of *Ms* Δ*gplH* + pCP0-*gplH* (not shown). The selective ion monitoring MS analysis of the mutant strain *Ms* Δ*gplH* revealed that the strain lacks the alditol acetate derivatives. For illustration clarity, only the *m/z* values of selected diagnostic molecular ions are indicated in the spectra. These molecular ions arise from the fragmentation patterns of the corresponding alditol acetates as displayed next to each spectrum.

The presence of GPLs was probed for in lipid samples from *Ms* WT + pCP0, *Ms* Δ*gplH* + pCP0, and *Ms* Δ*gplH* + pCP0-*gplH* (complemented strain) by GC-MS analysis as well. The pCP0-bearing strains, *Ms* WT + pCP0 and *Ms* Δ*gplH* + pCP0, rather than their respective plasmid-free parental strains, were used in these experiments so that the WT, the mutant, and the complemented strain could all be cultured under identical conditions (*i.e*., kanamycin-containing growth medium) for comparative analysis by GC-MS. Representative results from the GC-MS analysis are shown in Figure 6. This analysis probed for the presence of the alditol acetate derivatives of the characteristic glycosyl residues of *Ms* GPLs as a fingerprint indicator of the presence of GPLs in the lipid samples analyzed [47]. The GC-MS analysis of samples from *Ms* WT + pCP0 revealed the expected *m/z* peak array consistent with the characteristic presence of alditol acetate derivatives of the 2,3,4-trimethyl-rhamnose, 3,4-dimethyl-rhamnose and 6-deoxy-talose components of GPLs [7,8,47]. Conversely, these alditol acetate derivatives were not detected by GC-MS analysis of samples from *Ms* Δ*gplH* + pCP0. The samples from the complemented strain, *Ms* Δ*gplH* + pCP0-*gplH*, displayed an *m/z* peak array comparable to that of *Ms* WT + pCP0 and consistent with the presence of the alditol acetate derivatives originating from GPLs (not shown). Overall, the results of the GC-MS analysis and the results of the TLC analysis are in agreement with each other and, coupled with our genetic complementation-controlled analysis, conclusively demonstrate that *gplH* is essential for production of GPLs.

Based on the recent elucidation of the function of MbtH-like proteins as amino acid adenylation domain activators [36-40], it is reasonable to propose that GplH is required for the assembly of the D-Phe-D-*allo*Thr-D-Ala-L-alaninol core moiety of GPLs by the Mps1-Mps2 NRPS system. GplH might act as a critical activator of the amino acid adenylation activity of one or more of the four amino acid adenylation domains predicted by sequence analysis of the Mps1-Mps2 NRPS system [22,23]. Biochemical studies will be required to investigate this possibility.

### MbtH-mediated cross-talk between GPL biosynthesis and mycobactin biosynthesis

We noted that *Ms* has two potential *mbtH*-like genes located outside the GPL biosynthetic gene cluster. One of these genes is the *mbtH* orthologue in the mycobactin biosynthetic gene cluster of *Ms* mentioned above [35]. The second gene, MSMEG_0016, is clustered with genes implicated in the production of the siderophore exochelin [48-50]. The protein products of these two *Ms gplH* paralogues have considerable amino acid sequence identity between themselves and with GplH and *M. tuberculosis* MbtH (Figure 3B). The GPL deficiency of *Ms* Δ*gplH* indicates that neither of these two *Ms gplH* paralogues can support the production of GPLs in *Ms* Δ*gplH* to a meaningful level under our culturing conditions.

It is worth noting that *Ms mbtH* and MSMEG_0016 are associated with siderophore production pathways known to be repressed during growth under iron-rich conditions [51,52]. This fact raises the possibility that neither of these genes is expressed (or they are poorly expressed) in the iron-rich standard Middlebrook media used in our studies. With this consideration in mind, we explored whether an increase in expression of *Ms mbtH* (encoding the paralogue with the higher homology to GplH, Figure 3) could complement the GPL deficiency of *Ms* Δ*gplH*. To this end, we evaluated GPL production in *Ms* Δ*gplH* after transformation of the mutant with pCP0-*mbtH*Ms (expressing *Ms mbtH*). TLC analysis of lipid extracts from the transformant revealed the presence of GPLs, thus indicating that plasmid-directed constitutive expression of *Ms mbtH* complements the GPL deficient phenotype of *Ms* Δ*gplH* (Figure 5). Thus, it appears that *Ms* MbtH has the potential to functionally replace GplH if present in sufficient quantities. This cross-complementation phenomenon is in line with recent cell-based studies demonstrating MbtH-like protein-mediated cross-talk between NRPS systems [41,44]. Our finding is also consistent with reported *in vitro* enzymology indicating that, at least in some cases, the activity of amino acid adenylation domains of NRPSs can be stimulated not only by *bona fide* MbtH-like protein partners, but also by MbtH-like protein homologues from disparate natural product biosynthetic pathways [39,40].

### Deletion of *gplH* leads to a pleiotropic phenotype

**Figure 7:**
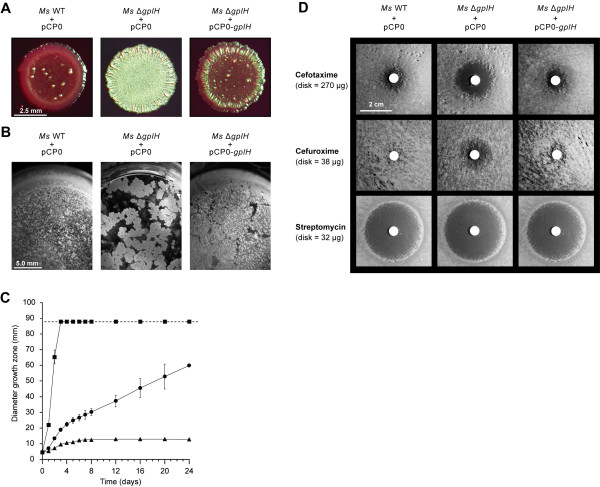
**Pleiotropic phenotype of *M. smegmatis *Δ*gplH.*** (**A**) Morphotype on the congo red agar plate assay. (**B**) Formation of biofilm at the liquid-air interface. **(C)** Sliding motility analysis. (▄) *Ms* WT + pCP0, (●) *Ms* Δ*gplH* + pCP0-*gplH*, (▲) *Ms* Δ*gplH* + pCP0. Data points are means of duplicates ± SEM. The dashed line marks the diameter of the agar plate. (**D**) Antimicrobial drug susceptibility. Results shown are representative of four determinations.

Colony morphotype, biofilm formation and sliding motility are properties that have been shown to be altered in GPL deficient mutants [18-20,23]. Loss of GPL also perturbs bacterial surface properties [19,32] and reduces the cell-wall permeability barrier to chenodeoxycholate uptake [19]. Interestingly, an altered profile of GPLs has been observed in drug-resistant MAC isolates [21]. This finding raises the possibility that GPL production might have an impact on antimicrobial drug susceptibility as well. We investigated whether deletion of *gplH* had an effect on all these properties. *Ms* WT + pCP0 and *Ms* Δ*gplH* + pCP0, rather than their respective plasmid-free parental strains, were used in the experiments so that the WT, the mutant, and the complemented *Ms* Δ*gplH* + pCP0-*gplH* strain could all be cultured under identical conditions (*i.e*., kanamycin-containing growth media) for comparative analysis. Representative results from these studies are shown in Figure 7.

*Ms* is known to develop into smooth, reddish colonies with a glossy and translucent appearance when grown on low carbon source congo red agar plates [23]. As expected, *Ms* WT + pCP0 displayed this characteristic morphotype in our congo red agar plate assay (Figure 7A). *Ms* Δ*gplH* + pCP0, however, had a drastically different morphotype. The mutant was characterized by rough, whitish colonies with a non-translucent and dried appearance. The strain *Ms* Δ*gplH* + pCP0-*gplH* had a morphotype more similar to WT than to that of the mutant, indicating partial complementation by episomal expression of *gplH* in the congo red agar assay. Deletion of *gplH* also altered the ability of *Ms* to form biofilms (Figure 7B). *Ms* WT + pCP0 formed a continuous, thin biofilm at the liquid-air interface, as expected based on previous reports [53,54]. In contrast, *Ms* Δ*gplH* + pCP0 failed to develop such a biofilm and instead grew as chunky patches on the liquid surface. The strain *Ms* Δ*gplH* + pCP0-*gplH* produced biofilms comparable to those seen with *Ms* WT + pCP0. Sliding motility was also compromised in *Ms* Δ*gplH* + pCP0 (Figure 7C). The mutant did not show sliding motility, whereas *Ms* WT + pCP0 was highly active in the motility assay. *Ms* Δ*gplH* + pCP0-*gplH* also displayed sliding motility, although the motility was somewhat reduced compared to WT. This observation indicates partial complementation by episomal expression of *gplH*. Overall, these results clearly indicate that deletion of *gplH* has a profound impact on colony morphotype, biofilm formation, and sliding motility. These mutant phenotypes have previously been associated with other GPL deficient strains and attributed to alterations of the properties of the cell surface due to lack of GPLs. Thus, it is likely that the phenotypes observed in the *gplH* mutant arise from its GPL deficiency.

In contrast to the drastic impact of the *gplH* deletion on colony morphotype, biofilm formation and sliding motility, lack of *gplH* had a relatively minor effect on antimicrobial drug susceptibility. When compared with *Ms* WT + pCP0 (control strain), *Ms* Δ*gplH* + pCP0 showed a slight, yet consistent, increase in susceptibility to only two drugs (cefuroxime and cefotaxime) from a panel of 15 drugs of different classes tested in standard disk diffusion assays.

Interestingly, these two drugs belong to the cephalosporin class, suggesting that the hypersusceptibility of the mutant is antibiotic-class dependent. Representative results illustrating the hypersusceptibility of the mutant to these cephalosporins are shown in Figure 7D.

Streptomycin susceptibility results are also shown in Figure 7D. The streptomycin susceptibility is presented as an example of those drugs to which the mutant had no meaningful difference in susceptibility relative to the WT control. The *Ms* Δ*gplH* + pCP0-*gplH* strain showed a drug susceptibility pattern similar to that of *Ms* WT + pCP0, indicating that the hypersusceptible phenotype of the mutant was complemented by episomal expression of *gplH*.

The molecular mechanism behind the cephalosporin hypersusceptibility arising from the lack of *gplH* remains obscure. It is generally believed that the permeability barrier imposed by the mycobacterial outer membrane reduces antibiotic susceptibility by decreasing compound penetration. Thus, it is tempting to hypothesize that the observed cephalosporin hypersusceptibility arises from an alteration in the permeability barrier of the outer membrane of the *gplH* mutant due to the lack of GPLs. The observation that lack of GPLs correlates with a reduction in the permeability barrier to chenodeoxycholate uptake [19] is in line with this hypothesis. The absence of GPLs might produce structural or fluidity changes in the membrane that lead to an increase in cephalosporin penetration. The fact that *Ms* Δ*gplH* displays only a modest increase in antibiotic susceptibility suggests, however, that the lack of GPLs in the outer membrane of the mutant does not have a profound effect on the permeability barrier that this cell envelope structure presents to drug penetration. Thus, our results support the view that GPLs are not critical contributors to the physical integrity of the permeability barrier of the mycobacterial cell envelope.

## Conclusions

Our results unambiguously demonstrate that the conserved gene *gplH* is required for GPL production and its inactivation leads to a pleiotropic phenotype. While genes encoding members of the MbtH-like protein family have been shown to be required for production of siderophores or antibiotics [41-44], our findings present the first case of one such gene required for biosynthesis of a cell wall component. Furthermore, *gplH* is the first *mbtH*-like gene with proven functional role in a member of the *Mycobacterium* genus. Altogether, this study formally demonstrates a critical role for *gplH* in mycobacterial biology and advances our understanding of the genetic requirements for the biosynthesis of an important group of constituents of the unique mycobacterial outer membrane.

## Methods

### Culturing conditions and recombinant DNA manipulations

*Ms* strain mc^2^155 (ATCC 700084) and its derivatives were routinely cultured under standard conditions (37°C, 225 rpm) in Middlebrook 7H9 (Difco) supplemented with 10% ADN (5% BSA, 2% dextrose, 0.85% NaCl), 0.2% glycerol and 0.05% Tween-80 (supplemented 7H9) or in Middlebrook 7H11 (Difco) supplemented with 10% ADN (supplemented 7H11) [55]. *E. coli* DH5α (Invitrogen) was cultured under standard conditions in Luria-Bertani media [56]. When required, kanamycin (30 μg/ml), hygromycin (50 μg/ml), sucrose (2%) and/or X-gal (70 μg/ml) were added to the media. General recombinant DNA manipulations were carried out by standard methods and using *E. coli* as the primary cloning host [56]. Molecular biology reagents were obtained from Sigma, Invitrogen, New England Biolabs, Novagen, QIAGEN, or Stratagene. Oligonucleotides were purchased from Integrated DNA Technologies, Inc. PCR-generated DNA fragments used in plasmid constructions were sequenced to verify fidelity. Chromosomal DNA isolation from and plasmid electroporation into mycobacteria were carried out as reported [55]. Table 1 lists the plasmids and oligonucleotide primers used in this study.

**Table 1:** Plasmids and oligonucleotide primers

**Plasmid**	**Characteristics**	**Source or Reference**
pCR2.1-TOPO	Cloning vector, kanamycin resistance and ampicillin resistance genes	Invitrogen
pCP0	Vector for gene expression in mycobacteria, kanamycin resistance gene	[4]
pCP0-*gplH*	pCP0 expressing *M. smegmatis gplH*	This study
pCP0-*mbtH*Ms	pCP0 expressing *M. smegmatis mbtH* (MSMEG_4508)	[35]
p2NIL	Kanamycin resistance gene and OriE	[57]
pGOAL19	Hygromycin resistance gene, *sacB*-*lacZ Pac*I cassette, and OriE	[57]
p2NIL-GOALc-ΔgplHc	Delivery vector carrying a *gplH* deletion cassette (ΔgplHc)	This study
**Oligonucleotide**	**Sequence (5’ to 3’)**	**Characteristics**
pepOF	GGTACCTGTTCAACGCGGCCAGAGCGTCATTGGTCTCGGCCA	*Kpn*I
pepOR	TTAATTAATGTTGCAACAGCTCCCTGATCCGGATGTCGACGTGCTTG	*Pac*I
pepIR	TCAGCCGTCAAGAGCAAAGCTGCCGTTGTCGTCATCGAACGGGTTGAT	SOE PCR
pepIF	CGACAACGGCAGCTTTGCTCTTGACGGCTGAGTCAAATAGTCTGTTG	SOE PCR
pepF	CTGCAGTGAACAGCCGGGAGAAACGT	*Pst*I
pepR	AAGCTTCCCAACAGACTATTTGACTCAGCCG	*Hin*dIII

### Construction of *M. smegmatis* Δ*gplH*

*Ms* Δ*gplH* was engineered using the p2NIL/pGOAL19-based flexible cassette method [57] as previously reported [4,31,35,58]. A suicide delivery vector (p2NIL-GOALc-ΔgplHc, see below) carrying a *gplH* (MSMEG_0399) deletion cassette (ΔgplHc) was used to generate *Ms* Δ*gplH*. The vector was electroporated into *Ms* and transformants with a potential p2NIL-GOALc-ΔgplHc integration via a single-crossover event (blue colonies) were selected on supplemented 7H11 containing hygromycin, kanamycin, and X-gal. The selected transformants were then grown in antibiotic-free supplemented 7H9, and subsequently plated for single colonies on supplemented 7H11 containing sucrose and X-gal. White colonies that grew on the sucrose plates were re-streaked onto antibiotic-free and antibiotic-containing supplemented 7H11 plates to identify clones that lost drug resistance, a trait indicating a possible double-crossover event with consequent loss of *gplH* or reversion to WT. The deletion of *gplH* in antibiotic sensitive clones was screened for and confirmed by PCR. Towards this end, chromosomal DNA isolated from mutant candidates was used as template along with primer pairs (pepOF and pepOR, pepF and pepR) that produced diagnostic amplicons permitting differentiation between the mutant and WT genotypes.

### Construction of p2NIL-GOALc-ΔgplHc and pCP0-*gplH*

The plasmid p2NIL-GOALc-ΔgplHc used in the construction of *Ms* Δ*gplH* carried the *gplH* deletion cassette ΔgplHc. The deletion cassette contained: 995-bp segment upstream of *gplH* + *gplH*’s first 13 codons (5 fragment) followed by *gplH*’s last 4 codons + stop codon + 1,000-bp segment downstream of *gplH* (3 fragment). ΔgplHc was built by the joining of the 5' fragment and the 3' fragment using splicing-by-overlap-extension (SOE) PCR [59]. Each fragment was PCR-generated from chromosomal DNA. Primer pair pepOF and pepIR and primer pair pepIF and pepOR were used to generate the 5’ and 3’ fragments, respectively. The fragments were then used as template for PCR with primers pepOF and pepOR to fuse the fragments and create ΔgplHc (2,061 bp). The PCR-generated ΔgplHc was first cloned into pCR2.1-TOPO (Invitrogen). ΔgplHc was subsequently excised from the pCR2.1-TOPO construct using *Kpn*I and *Pac*I, and the excerpt was ligated to p2NIL [57] linearized by *Kpn*I-*Pac*I digestion. The resulting p2NIL-ΔgplHc plasmid and plasmid pGOAL19 [57] were digested with *Pac*I, and the *Pac*I cassette (GOALc, 7,939 bp) of pGOAL19 was ligated to the linearized p2NIL-ΔgplHc to create p2NIL-GOALc-ΔgplHc. To create pCP0-*gplH*, the plasmid used for complementation analysis, a DNA fragment (266 bp) encompassing *gplH* and its predicted ribosome binding site (RBS) was PCR-amplified from genomic DNA with primer pair pepF and pepR and cloned into pCR2.1-TOPO. The RBS-*gplH* fragment was subsequently excised from the pCR2.1-TOPO construct using *Pst*I and *Hin*dIII and ligated to plasmid pCP0 [4] linearized by *Pst*I-*Hin*dIII digestion to create pCP0-*gplH*. The cloning placed *gplH* under the control of the *hsp60* promoter of pCP0 for gene expression in mycobacteria.

### Extraction and thin layer chromatography (TLC) analysis of GPLs

GPLs were extracted and analyzed by TLC by reported methods [22,60]. Cells from cultures (5 ml, OD_600_ of 1.3-1.6) grown in supplemented 7H9 as described above were collected by centrifugation (4,700 × g, 15 min), washed with cold phosphate buffered saline (PBS, 1 ml), and processed for GPL extraction. GPLs were extacted with 2:1 CHCl_3_/CH_3_OH (20 μl/mg wet weight) by incubation overnight at room temperature in a rocking shaker. After incubation, insoluble material was removed by centrifugation (18,400 × g, 10 min), and the CHCl_3_/CH_3_OH supernatant was recovered and mixed with 0.2 volumes of 0.9% NaCl. After vigorous vortexing, the mixture was centrifuged (1,150 × g, 5 min) and the organic phase (containing GPLs) was collected and evaporated to dryness. The dried lipid extracts were dissolved in 20 μl of CHCl_3_/CH_3_OH (2:1) and subjected to TLC using aluminum-backed, 250-μm silica gel F_254_ plates developed with CHCl_3_/CH_3_OH (100:7). After chromatography, TLC plates were sprayed with orcinol/sulfuric acid (0.1% orcinol in 40% sulfuric acid) and glycolipids were detected by charring at 140°C.

### Preparation and gas chromatography–mass spectrometry (GC-MS) analysis of alditol acetate derivatives

Alditol acetate derivatives of glycosyl units from GPLs were prepared and analyzed as reported [47,61]. Briefly, lipid samples prepared by extraction as noted above were acid-hydrolyzed in 250 μl of 2 M trifluoroacetic acid for 2 hr at 120°C. After cooling down to room temperature, samples were hexane-washed (250 μl) and dried on air bath after adding 1 μg of 3,6-*O*-dimethyl-glucose as an internal standard. The hydrolyzed sugars were reduced overnight at room temperature by adding 250 μl of NaBD_4_ (prepared at 10 mg/ml in 1 M NH_4_OH in C_2_H_5_OH). After reduction, glacial acetic acid (20 μl) was added to remove excess NaBD_4_ and the samples were dried. CH_3_OH (100 μl) was added to each sample, and after resuspension the solvent was evaporated to dryness (this step was repeated twice). The samples were per-*O*-acetylated with 100 μl of acetic anhydride at 120°C for 2 hr. After cooling, the samples were dried on air bath and suspended in 3 ml of CHCl_3_/H_2_O (2:1) by vortexing. The organic layer was extracted after centrifugation (2,500 × g, 5 min, 4°C) and dried on air bath. GC-MS analysis was performed using a Varian CP-3800 gas chromatograph (Varian Inc., Palo Alto, CA) equipped with a MS-320 mass spectrometer and using helium gas. The alditol acetate derivatives were dissolved in 50 μl of CHCl_3_ before injection on a DB 5 column (30 m × 0.20 mm inner diameter) with an initial oven temperature of 50°C for 1 min, followed by an increase of 30°C/min to 150°C and finally to 275°C at 5°C/min.

### Congo red agar plate assay

The assay was carried out using reported methodologies [23]. Briefly, mycobacterial cultures (5 ml, OD_600_ = 1.5) were shortly vortexed with glass beads to increase homogeneity and then centrifuged (4,700 × g, 15 min) for cell collection. The collected cells were washed with PBS (5 ml) and subsequently resuspended in PBS to an OD_600_ of 1. The cell suspensions were spotted (2 μl) on congo red agar plates [23] (7H9 basal medium, 1.5% agar, 100 μg/ml congo red (sodium salt of 3,3'-([1,1'-biphenyl]-4,4'-diyl)bis(4-aminonaphthalene-1-sulfonic acid), Sigma Aldrich Co.), 0.02% glucose, 30 μg/ml kanamycin). Colony morphology was examined using an Olympus SZX7 stereo microscope after plate incubation (37°C, 3 days).

### Sliding motility test

The test was performed by standard methods [19]. Briefly, mycobacterial cell suspensions in PBS prepared as noted above were spotted (2 μl) on sliding motility assay plates [19] (7H9 basal medium, 0.3% agarose, 30 μg/ml kanamycin). The sliding motility plates were incubated at 37°C and the degree of spreading (diameter of growth zone) was determined at the time points indicated in results.

### Biofilm formation assay

The liquid-air interface biofilm assay was conducted based on reported methods [53]. Overnight mycobacterial cultures (5 ml) grown in supplemented 7H9 as noted above were centrifuged (4,700 × g, 15 min) for cell collection. The cells were washed twice with 5 ml of supplemented 7H9 without Tween-80. After washing, the cells were resuspended in supplemented 7H9 without Tween-80 to a calculated OD_600_ of 10. A 25 μl aliquot of each suspension was inoculated onto the surface of 2.5 ml of supplemented 7H9 without Tween-80 loaded into a well of a 12-well polystyrene plate. The plate was incubated for 4 days without shaking at 37°C before examination for biofilm formation.

### Drug susceptibility assay

Standard disk-diffusion assays were carried out as reported [58,62]. Exponentially growing cultures (OD_600_ = 0.6) in supplemented 7H9 were diluted in fresh medium to an OD_600_ of 0.05, and 100 μl of diluted culture were used to seed 7H11 plates (20 ml agar/plate). Antibiotic disks were placed onto the inoculated agar and the plates were incubated at 37°C for 2 days before analysis. The antibiotics tested were doxycycline, isoniazid, streptomycin, tetracycline, cefuroxime, erythromycin, ciprofloxacin, levofloxacin, ethambutol, ethionamide, rifampicin, clarithromycin, cefuroxime, cephalexin, and cefotaxime. The antibiotics were acquired from Sigma-Aldrich, Fisher Scientific, Tokyo Chemical Industry, or Calbiochem Biochemicals.

## Abbreviations

GC-MS: Gas chromatography–mass spectrometry; GPL: Glycopeptidolipid; MAC: *Mycobacterium avium-Mycobacterium intracellulare* complex; *Ms*: *Mycobacterium smegmatis*; NRPS: Nonribosomal peptide synthetase; PBS: Phosphate buffered saline; PKS: Polyketide synthetase; TLC: Thin layer chromatography.

## Competing interests

The authors declare that they have no competing interests.

## Authors’ contributions

LQ conceived the study. ET, SC, PM, UE and SA carried out the experiments. LQ, ET, SC, and DC analyzed results and drafted the manuscript. All authors read and approved the final manuscript.
